# Actioning the findings of hard endpoint clinical trials as they emerge in the realm of chronic kidney disease care: a review and a call to action

**DOI:** 10.1093/ckj/sfae035

**Published:** 2024-02-09

**Authors:** Giovanni F M Strippoli, Suetonia C Green

**Affiliations:** Sydney School of Public Health, The University of Sydney, NSW Australia; Department of Precision and Regenerative Medicine and Ionian Area (DIMEPRE-J) University of Bari Aldo Moro, Bari, Italy; Department of Medicine, University of Otago, Christchurch, Christchurch, New Zealand

**Keywords:** clinical trial, CONVINCE, epidemiology methods, evidence-based nephrology, hemodiafiltration, systematic review

## Abstract

Fewer than half of patients treated with hemodialysis survive 5 years. Multiple therapeutics are used to address the complications of advanced chronic kidney disease but most have not been found to improve clinical outcomes. Clinical trials of treatment innovations for chronic kidney diseases and dialysis care have been suboptimal in number and quality. Recent trials are changing this trend. Practice and policy change when new evidence emerges remains frequently impeded by resource and organizational constraints and accordingly, clinical practice guidelines are updated years or decades after definitive evidence is produced. Ultimately, practice change in health systems is slow, leading to impaired uptake of effective medical interventions and lower value healthcare, although innovations in rapid guideline production are emerging. What can be done to ensure that conclusive evidence is taken up in practice, policy and healthcare funding? We use the example of the recently published hard endpoint study “Comparison of high-dose HDF with high-flux HD” (CONVINCE) (hemodiafiltration versus hemodialysis), to explain how a new trial can impact on medical knowledge and change in practices. We (i) assess how the trial can be placed in the context of the totality of the evidence, (ii) define whether or not further trials of convective dialysis therapies are still needed and (iii) examine whether the evidence for convective therapies is now ready to inform practice, policy and funding change. When looking at CONVINCE in the context of the totality of evidence, we show that it addresses dialysis quality improvement priorities and is consistent with other trials evaluating convective dialysis therapies, and that the evidence for convective dialysis therapies is now definitive. Once updated evidence for cost-effectiveness in specific healthcare settings and patient-reported outcomes become available, we should therefore determine whether or not clinical practice guidelines should recommend uptake of convective dialysis therapies routinely, and move on to evaluating other treatments.

## THE CHALLENGE OF CLINICAL OUTCOMES IN PATIENTS WITH ADVANCED CHRONIC KIDNEY DISEASE

Over half of people commencing dialysis are not alive at 5 years and direct costs of dialysis care surpass 15 billion EUR each year in Europe alone [1]. Globally, dialysis care is insufficiently funded to treat all people with advanced chronic kidney disease who would benefit [2]. The symptoms experienced by people with advanced chronic kidney disease are severe and difficult to alleviate [3]. Kidney transplantation, as the gold standard treatment option, is not universally available [4].

People who have advanced chronic kidney disease develop coexisting conditions including vascular disease and infection, which herald a rapid decline in health [5]. These complications render the impact of clinical treatments to be of negligible benefit [6], likely due to the different pathobiology of vascular disease compared with the general population and competing coexisting medical conditions. The heterogeneous phenotypes of chronic kidney diseases impede bench-to-bedside development of disease-modifying therapeutics. While the cellular mechanisms of many genetic conditions including focal and segmental glomerulosclerosis [7] and polycystic kidney diseases [8] have been elucidated, this has not rapidly led to parallel breakthroughs in disease prevention in the way that transformative outcomes for cystic fibrosis have been achieved [9]. An additional layer of complexity in the pursuit of evidence arises when considering kidney replacement therapies, as they involve intricate medical device–based modalities where practice patterns may vary. Patient outcomes in this context may result from a combination of therapy efficacy, the quality of the care delivery and patient adherence.

## CURRENT STATE OF THE ART OF CLINICAL TRIALS IN CHRONIC KIDNEY DISEASE

The randomized clinical trial is the gold standard for evaluating therapeutics, procedures and devices. There has been historically suboptimal evidence in kidney care [10], with clinical trials in nephrology often including a small number of participants and follow-up of treatment effects for only weeks to months [11]. Added to this, trials in dialysis care are nearly universally neutral (providing no evidence of benefit)—with a legacy of promising therapeutics ultimately showing no effectiveness [12–18]. It should be commended that the nephrology research community continues to respond with ever larger and more definitive clinical trials completed (PIVOTAL [19]) or underway (SIMPLIFIED [20], RESOLVE [12], PHOSPHATE [13], HiLo [14]). Notably, trials of sodium-glucose cotransporter 2 (SGLT2) inhibitors and glucagon-like peptide-1 (GLP1) receptor agonists are also changing the trajectory of evidence in kidney diseases [15, 16].

## THE CHALLENGES OF CHANGING PRACTICE AND POLICY FOLLOWING NEW EVIDENCE

Translation of hard endpoint evidence directly and rapidly into practice and policy is slow and inconsistent [17]. Despite four trials showing that streptokinase reduced death from myocardial infarction by 25% in 1971, it took another 17 years, 29 further trials and the allocation of 20 000 people to placebo before streptokinase became routine care [18]. This example eloquently shows how a poorly developed evidence translation environment can lead to inadequate treatment of a common and highly fatal condition for two decades. Why did this knowledge translation failure occur?

First, it has only been recent that hard endpoints for clinical trials in chronic kidney diseases have been standardized—the Standardised Outcomes in Nephrology (SONG) [21]. Standardization is a crucial step in ensuring that clinical trials report hard endpoints and that these can be meaningfully aggregated to understand the evidence wherever possible.

Second, evidence from clinical trials must be considered in their totality. It is frequently observed that a large new trial is a focus of policy or practice change or debate. For example, one of the largest trials ever conducted in people with kidney diseases—the Study of Heart and Renal Protection (SHARP) trial—concluded that lowering cholesterol with simvastatin plus ezetimibe safely reduced major atherosclerotic events in a wide range of patients with advanced kidney disease, including those treated with dialysis [22]. A focus on this as a single trial led to UK and global guidelines recommending statin therapy in dialysis patients [23]. When the evidence was subsequently reviewed in its totality a year later there was high-quality evidence that statin therapy had little or no effect on the risks of death in people on dialysis [24]. The reverse is also observed—that the effects of a treatment in a subgroup of a trial population are interpreted as the key finding to support practice limitations or change. For example, a smaller effect of aspirin was observed in women in subgroup analyses of trials of coronary heart disease, leading to the belief that women did not derive the same benefit of treatment as men, and were undertreated as a direct result [25].

Third, while there is considerable resource investment in clinical trials, the same cannot be said for evidence synthesis activities and guidelines, which are the next steps in the policy and reimbursement evidence chain. The median estimated cost of a trial for an Food and Drug Administration–approved therapeutic was USD $19 million [26]. The leading global evidence synthesis organization, Cochrane, by contrast, had funding in 2020 of approximately £30 million. The disparity in investment is stark and leads to a highly inefficient evidence ecosystem beyond the existing fragility of individual trials [27]. The emergence of artificial intelligence and computable evidence synthesis are promising to the reduce delays in evidence synthesis but are not yet prime time or having an impact on robust evidence production [28].

Fourth, clinical practice guidelines are essential in knowledge translation from clinical trials to patient care. Guidelines integrate not just evidence from all clinical trials and priority patient outcomes, but also include patient preferences, cost-effectiveness considerations and health justice, together with how certain we are in the evidence. Guidelines frequently do not change practice, or change is slow for the very reason that individual health workers are tasked with their implementation independent from systemic change at a healthcare provider, policy or funder level. This suggests that to effect change our guideline programs should be directly connected to regulatory, policy and reimbursement systems [29].

## A RECENT EXAMPLE OF A HARD ENDPOINT TRIAL IN HEMODIALYSIS AND HOW CAN WE PROPERLY RESPOND TO INFORM QUALITY IMPROVEMENT OF PRACTICE, POLICY AND REIMBURSEMENT

**Figure 1:**
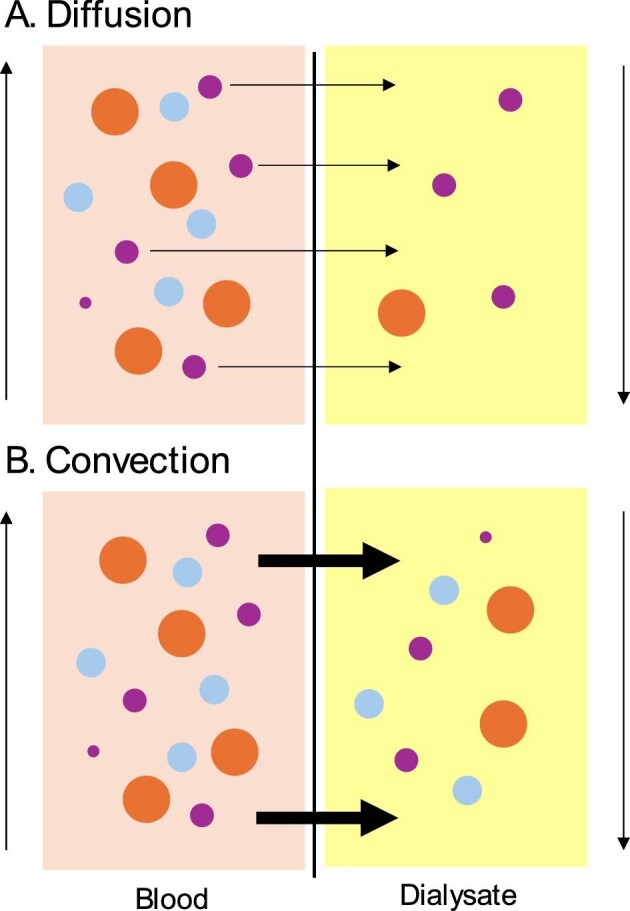
The process of diffusion and convection in removal of uremic solutes during kidney replacement therapy. Hemodiafiltration and hemofiltration are convective dialysis therapies. (**A**) Diffusion occurs across a membrane when molecules move down the concentration gradient (in this case, from the blood stream of the patient down the gradient into the dialysate fluid). Larger molecules cross the membrane more slowly and smaller molecules are cleared to a greater extent. (**B**) Convection is where uremic solutes are dragged across the dialysis membrane into the dialysis waste with fluid flow generated by a pressure gradient across the membrane (the transmembrane gradient). Larger molecules are dragged across the membrane more rapidly than during diffusion alone, leading to greater clearance of larger uremic solutes.

The Comparison of high-dose HDF with high-flux HD (CONVINCE) trial comparing hemodiafiltration with high flux hemodialysis is the most recent landmark trial to be released for kidney failure [30]. Hemodiafiltration is a convective-based kidney replacement therapy (Fig. 1) that leads to greater removal of a larger uremic solutes in people with advanced chronic kidney disease than a diffusion therapy such as hemodialysis [31]. As an excess accumulation of uremic compounds is associated with poorer patient outcomes, convective-based therapy is a plausible dialysis advance to improve patient survival and wellbeing [32].

Prior to the CONVINCE trial, evidence for hemodiafiltration was deemed insufficiently certain to change established practice [33]. In the CONVINCE trial, 1360 patients with kidney failure were randomly allocated to receive either high-dose hemodiafiltration or high-flux hemodialysis with a primary outcome of death from any cause. After a median follow-up of 30 months, the risk of death from any cause (the primary outcome) was lower with high-dose hemodiafiltration than high-flux hemodialysis [hazard ratio (HR) 0.77; 95% confidence interval (CI) 0.65–0.93]. By contrast, hemodiafiltration did not significantly reduce death from cardiovascular causes (HR 0.81; 95% CI 0.49–1.33). Counter to the general dialysis population, cardiovascular deaths accounted for only one-quarter of the deaths. Three-quarters of the deaths in the CONVINCE trial, conducted during the coronavirus disease 2019 (COVID-19) pandemic, were attributable to infection, and most frequently to COVID-19. Accordingly, the trial may have been underpowered statistically to provide a definitive estimation of the risks of cardiovascular death with convective therapies.

Since the release of CONVINCE, there have been several articulate and detailed responses to the findings [34–39]. Key ideas related to CONVINCE by opinion leaders have included:

(1) The high volume of hemodiafiltration required as an entry criterion led to a relatively healthier population in which high volume hemodiafiltration was beneficial, so the results may not be generalizable to the wider dialysis population.
(2) The lack of evidence of reduced cardiovascular mortality with hemodiafiltration was surprising as the potential primary cause of action of high-dose HDF and indicates that the CONVINCE trial deviates from prior evidence. The opinion pieces raised the possibility of internal validity issues including death from cardiovascular causes misclassification related to COVID-19.
(3) The results of CONVINCE are insufficient to drive widespread adoption based on only a reduction in death from all causes and an uncertain mechanism of effect.
(4) The effects of hemodiafiltration differed between patients who had an arteriovenous fistula and those who did not, those with and without diabetes, and those with and without previous cardiovascular disease.
(5) The results of hemodiafiltration on health-related quality of life and cost effectiveness are awaited.
(6) The results of the currently ongoing High-volume HDF versus High-Flux HD Registry Trial (H4RT) with 1550 patients are required to understand whether hemodiafiltration is universally beneficial for people with advanced chronic kidney disease, including those with coexisting cardiovascular disease or diabetes [40].

**Figure 2:**
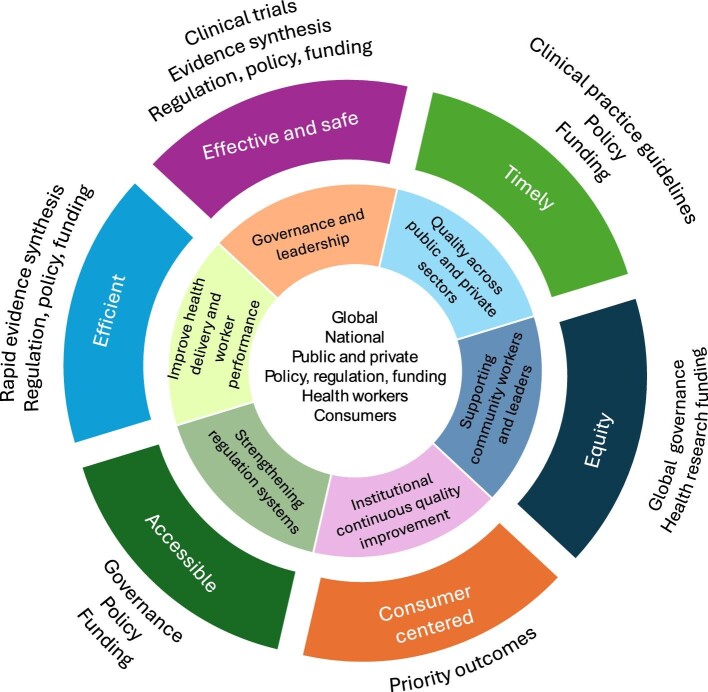
Continuous health system quality related to dialysis care. Continuous quality improvement in dialysis care requires an all-of-system approach based on knowledge translation aligned to key quality indicators (effective and safe, timely, equity, consumer centered, accessible and efficient). The concept of continuous improvement is recommended to support the generation of systems and governance that achieve healthcare quality. Examples in dialysis care include SONG-HD, global clinical practice guidelines and hard endpoint clinical trials. Global equity and accessibility activities include the International Society of Nephrology Global Kidney Health Atlas, and the EU-funded ERA4Health Partnership focusing on equity in public healthcare systems.

**Figure 3:**
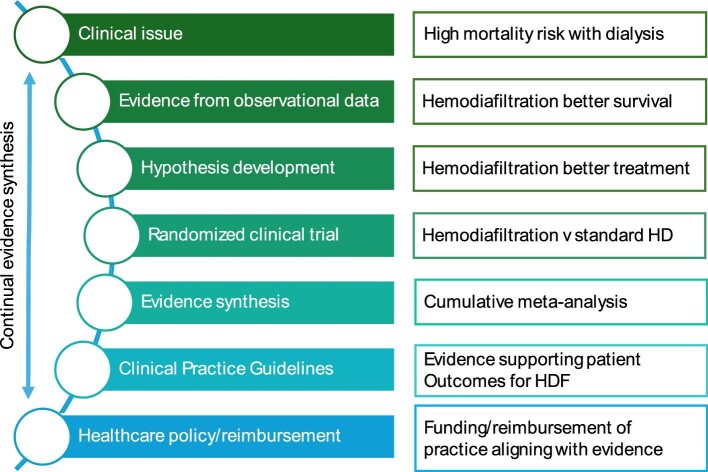
Suggested framework of changing regulation, practice and policy following new evidence, using the example of hemodiafiltration. HD, hemodialysis. HDF, hemodiafiltration. Framework of evidence translation into clinical practice, using hemodiafiltration for kidney failure as an example. The evidence process starts with a clinical issue considered important by patients, caregivers, health professionals and policymakers, such as the high mortality associated with kidney failure. Data drawn from dialysis registries and non-randomized studies indicates that hemodiafiltration is associated with better survival, but whether HDF is causal to better health outcomes is unknown. This generates the hypothesis to be tested that HDF improves survival compared with standard hemodialysis, which forms the basis for a randomized clinical trial (CONVINCE) [30]. We propose then that the results of the clinical trial are cumulated to enable the totality of the evidence from all related clinical trials to underpin knowing about our certainty of the evidence. This evidence and its related certainty inform timely Clinical Practice Guidelines developed using best practices, which in turn enable healthcare policy and reimbursement changes to support changing clinical practice at the bedside.

In this review, we use this example of the emergence of a new hard endpoint trial of hemodiafiltration and commentaries raising ideas about potential differences in the results of CONVINCE compared with previous trials to explore how the CONVINCE trial delivers health system quality improvement (Fig. 2) through regulation, reimbursement and policy settings based on a knowledge translation process (Fig. 3). That is, we explore how the review of this trial in the context of the totality of the existing trial evidence should inform any changes in practice and explore the issues raised by the nephrology community in published responses.

### Step 1: is the clinical issue of convective therapy of importance to patients, health workers, policy makers, regulators and health service providers

Based on SONG Hemodialysis (SONG-HD), the primary and secondary outcomes of CONVINCE match the critically important core outcomes for trials in hemodialysis for patients, caregivers, healthcare workers and policymakers—fatigue, cardiovascular disease, vascular access and mortality [21]. However, it is not clear how governments and dialysis for-profit companies might use the CONVINCE data for quality improvement. Examining the utility of the CONVINCE trial results on the broader priorities of health funding and health systems is harder as documentation and articulation of these priorities are less direct. They may be described in strategic documents or related funding body statements. For hemodialysis, we could consider, for example, the EU Global Health Strategy which includes the priority action of ensuring “that innovative vaccines, treatments and diagnostics for new, prevalent or neglected infections and non-communicable disease are developed and used, including through funding from Horizon Europe…” [41]. While not directly stating that hemodialysis care is part of this priority, it might be inferred as being an innovative treatment for non-communicable diseases as per the Strategy. The CONVINCE trial was funded by the EU Horizon 2020 program, which is the enactment of the European Commission's policy priorities to make a “real and sustainable difference to the quality of life in the EU.”

We might conclude from these stated priority settings that the results of the CONVINCE trial—through establishing the effectiveness of a dialysis therapy on patient health—can inform quality improvements in complex healthcare for kidney failure, as a non-communicable disease with a heavy burden on patients and societies.

### Step 2: what is the existing evidence on whether convective-based hemodialysis is more effective than diffusive hemodialysis?

The next step is to summarize the current evidence related to convection-based dialysis therapy prior to the CONVINCE trial. The most recent Cochrane systematic review summarizing the evidentiary basis for convective hemodialysis treatments included all trials published up to February 2015 [42]. The review summarized 40 randomized clinical trials in 3488 participants including all four major trials prior to the CONVINCE trial [42–46]. As of early 2015, and based on these trials, there was no evidence that hemodiafiltration reduced death from any cause in adults with kidney failure. The point estimate suggested a potential benefit of lower risk of death with hemodiafiltration, while the confidence interval—the interval of risks in which we are 95% confident the actual treatment effect exists—included the possibility of no effect (relative risk 0.87, 95% CI 0.72–1.05). Furthermore, the risks of bias in the available trials resulted in low certainty in the evidence (see Box 1), generating the rationale for the CONVINCE trial.

Also in that Cochrane review, there was evidence that hemodiafiltration reduced death from cardiovascular causes (the relative risk of cardiovascular death with convective-based dialysis therapy was 0.75 of the risk with diffusive hemodialysis, with a 95% CI of 0.66–0.80).

### Step 3: what does the new trial add to the existing evidence?

**Figure 4:**
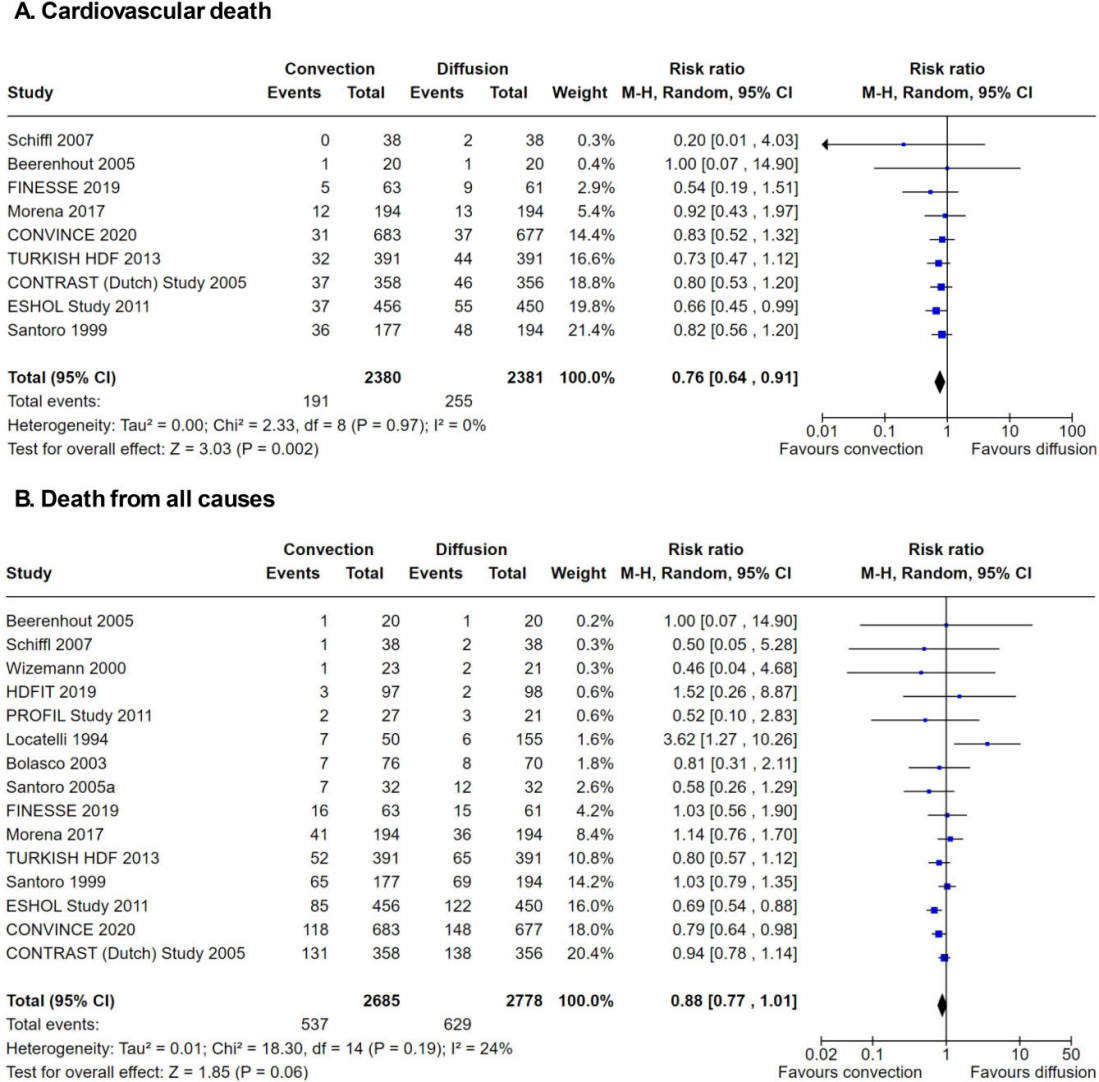
Risks of death due to cardiovascular cause and all causes with convective therapy versus diffusive therapy in adults with kidney failure. Forest plot of analyses of risks of (**A**) death due to cardiovascular causes and (**B**) death due to all causes in randomized controlled trials comparing convective therapies with diffusive therapies in adults with kidney failure. The I^2^ index estimates the percentage of variation between studies due to heterogeneity rather than chance. M-H: Mantel–Haenszel. The weight of the study refers to the amount of information they contribute. This gives studies with more precise results more weight in the overall effect.

New trials of convective-based kidney replacement therapies have been published since 2015. We conducted an updated search of the Cochrane CENTRAL electronic database on 31 August 2023. Overall, by 2023, 15 clinical trials reported death from any cause and 9 trials reported death from cardiovascular causes during treatment with convective compared with diffusive therapies for kidney failure (Fig. 4).

When summarizing the existing trials including CONVINCE, convective therapies reduced cardiovascular death (relative risk of 0.76 with a 95% CI 0.64 to 0.91) when compared with diffusive hemodialysis treatments. There was no evidence that the effects of convective therapy on death from cardiovascular causes varied across the studies. In other words, the trials found statistically similar results for the impacts of convective-based therapy, regardless of their participant populations, treatment practices or trial design. This means that the results of the CONVINCE trial are entirely consistent with the overall scientific evidence for convective-based therapies—that they reduce cardiovascular death by about 25% in people with kidney failure.

Box 1. GRADE certainty ratings.

**Certainty   What it means**
Very low   The true effect is probably different from the estimated effect
Low   The true effect might be markedly different from the estimated effect
Moderate   The true effect is probably close to the estimated effect
High   The true effect is similar to the estimated effect with high confidence

GRADE:   Grading of Recommendations, Assessment, Development, and Evaluations.

In absolute terms and based on an annual risk of cardiovascular death in people with kidney failure of 7%–8%, convective-based therapies for advanced chronic kidney disease might be expected to prevent one to two deaths per year among 100 treated patients [1].

Notably, in the totality of the evidence, death from any cause is not lowered with certainty with convective dialysis therapy compared with standard hemodialysis, including the CONVINCE trial (Fig. 4). Although the CONVINCE trial as a single trial identified lower mortality with convective compared with diffusive-based therapies, the overall evidence would suggest there is a smaller and possibly no benefit. There is also no evidence that the CONVINCE trial's result is statistically different from the other available studies.

An important critique of the CONVINCE trial is that the findings may not be applicable to the general dialysis population (generalizable) or may be limited to the healthier dialysis population (patients without diabetes or coexisting cardiovascular disease) [38, 39]. Indeed, the primary publication of the CONVINCE trial presents analyses of the primary outcome (death from any cause) and risk estimates in specific trial population subgroups (those with or without diabetes, those with or without cardiovascular disease and those with a permanent arteriovenous fistula as dialysis access, among other subgroups). The significant reduction in the risk of all-cause death observed with convective therapy appears limited to patients without diabetes or without cardiovascular disease, and particularly among those with an arteriovenous fistula. For example, the relative hazard of death among those without diabetes was 0.65 (95% CI 0.48–0.87) and the relative hazard in those with diabetes was 0.97 (95% CI 0.72–1.31). That these relative risk estimates appear different in their significance in subgroups is not sufficient to conclude that the results are only applicable to these specific subpopulations (without diabetes, without cardiovascular disease, with a fistula). In formal analysis, one should conduct a statistical test of interaction of the effect with the subgroup characteristic to evaluate statistically whether any subgroup difference really exists. An absence of effect may be due either to a true lack of subgroup effect or to low power in the subgroup analyses [47].

The question of whether benefits are restricted to specific patients can additionally be explored looking at the totality of evidence either by individual patient data meta-analysis or a study-level meta-regression. In such a metaregression of all available trials of convective versus diffusive therapy, there is no evidence that the risk of cardiovascular death is associated with the presence of diabetes, acknowledging the low statistical power of this analysis (Supplementary data, Fig. S1). Similar findings are observed for the presence of cardiovascular comorbidity or an arteriovenous fistula. In an individual patient data meta-analysis, conducted in 2016, there was no evidence that the comparative effects of hemodiafiltration compared with hemodialysis was different in subgroup analyses. While there may be biological reasons to justify potential lack of effect in people with diabetes in a trial like CONVINCE, the totality of evidence from all existing experimental studies (as shown in the meta-regression analysis) is not demonstrating such difference. The power of such analysis may be low, but empiric evidence of effect modification (meaning that an intervention is effective in a specific group of individuals and not in another, like for the example of statins, which have been proven to be effective in a large variety of populations, but not effective in people on hemodialysis) is a very rare event. At this moment in time it is also very unlikely that another properly sampled randomized trial can be conducted solely in a selected population (e.g. only people with diabetes). Based on these considerations, in the absence of evidence of harm and given the overall benefits found in the total body of evidence, there would be no strong reasons to advocate for treating people with diabetes differently from those without.

### Step 4: what does the existing evidence for convective therapies mean for clinical care, funding and policy?

Clinical practice guidelines incorporate the treatment effects from clinical trials, the certainty of the evidence, stakeholder preferences, the balance of benefits and harms, economic analyses and equity [29]. The Kidney Disease Outcomes Quality Initiative (KDOQI) concluded in 2015 that there was insufficient evidence for adoption of hemodiafiltration and that further research was needed before convective therapies could be recommended. In the evidence review for convective therapies versus diffusive therapies conducted by the National Institute for Health and Care Excellence (NICE) in the UK in 2017, the available clinical trials were considered to have potentially serious limitations and no recommendation for convective therapy was made [48].

To update the clinical guidelines, we need additional analyses of the effectiveness of convective therapy on cost-effectiveness and health-related quality of life. In our previous Cochrane review, the evidence for patient-reported quality of life and symptoms was of very low certainty or absent, consistent with another systematic review published in 2018 [49]. A summary of cost-effectiveness across the current studies in a range of clinical settings and countries, including low- and middle-income countries, would also assist to tailor guidelines in specific health systems and payer structures. Based on the current evidence summaries, it is not possible to make recommendations or infer the appropriate clinical care response to recent trials of convective therapies. The results of such analyses from the CONVINCE trial are awaited.

### Step 5: what is the next step—are more trials of convective therapies or should we focus on additional promising therapies?

Can we stop conducting additional trials comparing hemodiafiltration with diffusive hemodialysis? To assist this process, we can review the certainty of the current evidence. For death from any cause with convective-based therapy, the evidence certainty is moderate. This indicates that convective-based therapy probably has little or no effect on death from any cause among people with kidney failure. For cardiovascular death, the evidence is similarly of moderate certainty, indicating that convective-based therapies probably decrease cardiovascular death.

**Figure 5:**
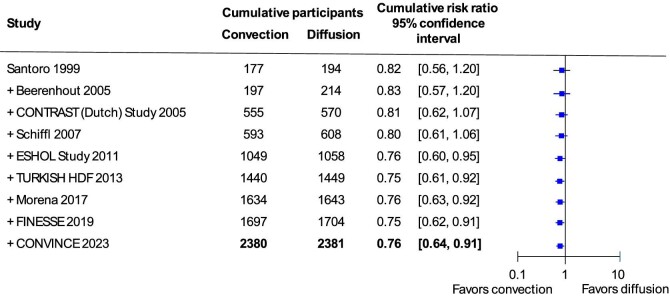
Cumulative meta-analysis of convective therapy on risks of cardiovascular mortality. The relative risk of cardiovascular death with convective versus diffusive therapy when subsequent trials are added sequentially to the summarized effects of all prior trials. The effect of convective therapy on cardiovascular mortality became evident in 2011 after approximately 2000 patients participated in five clinical trials. The involvement of an additional 2500 participants in four additional trials has provided similar results.

When the trial effects are cumulated over time, the evidence is now very consistent. Evidence of lowering cardiovascular mortality was first observed in 2011, and subsequent trials confirmed this finding over the next 12 years (Fig. 5). Based on the homogenous results in available trials and the stable cumulative effect of convective therapy with additional trials since 2011, it might be suggested that additional trials of hemodiafiltration compared with hemodialysis may not be informative.

Given the priority of innovative therapies indicated by the European Global Health Strategy, it is clearly necessary to conduct more trials of interventions to lower all-cause and cardiovascular mortality and address priority patient outcomes in people with kidney failure. In the dialysis realm there are so many dialysis treatments warranting large definitive clinical trials that do not yet have definitive evidence, including mid-cut-off membrane hemodialysis [50], intensified hemodialysis (longer hours or more frequent, or both) [51] and the use of artificial intelligence to generate treatment effects of randomization from existing non-randomized data [52].

## CONCLUSION

In conclusion, the totality of the evidence suggests that the CONVINCE trial is consistent with all existing trials comparing convective based versus diffusive therapies for people with kidney failure. The existing evidence for convective based therapies suggests that it offers a 25% reduction in cardiovascular death compared with diffusive-based therapies in people with advanced kidney disease, although there are limited data regarding patient-reported outcomes, cost-effectiveness and other features of health system quality improvement including equity and accessibility that warrant further evaluation. There is no evidence at present that the effects of convective therapy are different in patients with or without coexisting conditions or permanent fistula vascular access, and that the results of CONVINCE are generalizable. Given that the evidence for convective therapy is now moderate to high certainty, future trials of convective dialysis compared with standard hemodialysis are not required. We should proceed with conducting trials that evaluate other dialysis interventions such as medium cut-off membranes and intensified hemodialysis. Our patients deserve additional effective and proven treatments, and these trials will contribute to expanding our understanding and improving their care.

## Supplementary Material

sfae035_Supplemental_File

## Data Availability

The data underlying this article are available in the article and in its online Supplementary data.
