# Supplementary material for: Actioning the findings of hard endpoint clinical trials as they emerge in the realm of chronic kidney disease care: a review and a call to action
Source: Clin Kidney J. 2024 Feb 9;17(2):sfae035. doi: 10.1093/ckj/sfae035 (PMC10903297; doi:10.1093/ckj/sfae035)
Supplement: sfae035_Supplemental_File [file sfae035_supplemental_file.docx]

**Supplementary Figure 1 Meta-regression of the log risk ration for death from cardiovascular causes against the percentage of patients with a diagnosis of diabetes mellitus at baseline comparing hemodiafiltration with hemodialysis**

The proportion of variation in the risk ratio of cardiovascular death comparing hemodiafiltration with hemodialysis explained by the proportion of participants with diabetes is 0% (tau-squared 0.0000).(43,44,46,53–56) The heterogeneity between studies in relative risk of death from cardiovascular causes is 0% (tau squared = 0.00, I squared = 0%) (Figure 4). The proportion of participants with diabetes at baseline in the contributing trials ranged from 19.7% to 38.6%.
